# Computational Strategy for Analyzing Effective Properties of Random Composites–Part III: Machine Learning

**DOI:** 10.3390/ma18245531

**Published:** 2025-12-09

**Authors:** Vladimir Mityushev, Piotr Drygaś, Łukasz Walusiak

**Affiliations:** 1 Faculty of Computer Science and Mathematics, Cracow University of Technology, Warszawska St., 24, 31-155 Krakow, Poland; piotr.drygas@pk.edu.pl; 2 Faculty of Architecture, Civil Engineering and Applied Arts, The Department of Computer Science, Academy of Silesia, 43 Rolna Street, 40-555 Katowice, Poland; lukasz.walusiak@akademiaslaska.pl

**Keywords:** random composites, effective elastic constants, classification of dispersed microstructures, machine learning in composites

## Abstract

This paper continues the analysis from Parts I and II, which addressed two-dimensional dispersed random composites. This part extends previous analytical studies by incorporating machine learning (ML) methods to quantitatively classify microstructures. The methodology relies on decomposing the expressions for the effective tensors into geometrical and physical parts, represented by structural sums and component-specific physical constants. The study concerns a two-phase composite with non-overlapping circular inclusions embedded in an isotropic elastic matrix. The random distribution of inclusions ensures macroscopic isotropy of the composite. A key outcome is the explicit demonstration of how the effective tensor depends on the geometric probabilistic distributions of inclusions and the computational protocols employed in their realization. These steps constitute the strategy for studying elastic fibrous composites, classifying them by macroscopic properties, and describing an analytical algorithm to derive expressions for computing the effective constants. The decomposition theorem and the construction of feature vectors consisting of structural sums are used as inputs to the ML analysis. As a result, we develop a computationally effective strategy to classify dispersed random composites indistinguishable by simple observations.

## 1. Introduction

A dispersed random composite is an important class of material science. Classification of composites by reinforcement geometry in the engineering sciences is based on observation of their structures and determination of geometric parameters, such as concentrations of phases, shapes of inclusions, their sizes, correlation functions, etc. [1,2]. Unidirectional fiber-reinforced composites [3,4,5] constitute a class of two-dimensional (2D) dispersed composites (In the present paper, we give different meanings to the term “2D” referring to a section of composite perpendicular to fibers and “two-dimensional” concerning features of ML). Determining their macroscopic properties is the primary challenge of the homogenization theory [6,7], and its constructive implementation [8,9,10]. In the present paper, Part III, we utilize the analytical results of constructive homogenization obtained in [11,12], referred to as Parts I and II, respectively, to extend the study of two-phase fiber-reinforced composites using machine learning (ML) methods.

**Figure 1:**
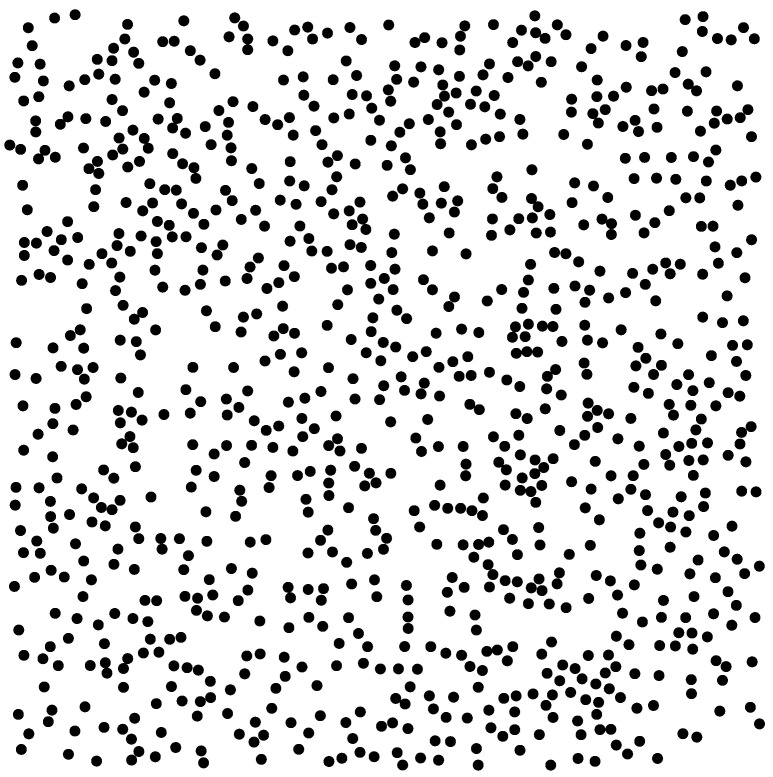
RVE including of 1000 disks.

We are interested in random composites and their microstructure. A fiber-reinforced composite is represented by its section perpendicular to the unidirectional fibers, more precisely, by a distribution of non-overlapping disks in a periodicity cell, identified with the Representative Volume Element (RVE) shown in Figure 1.

Here, we follow the revised Hill’s conception developed in Part II. It is worth noting that there exists an infinite many probabilistic distributions of non-overlapping disks [13], not only the unique uniform distribution (i.i.d. random variables) tacitly considered in the majority of published works [14].

Let the image analysis of composites be performed, and we proceed to discuss the next step of its interpretation. Having at their disposal pictures of microstructure, engineers may intuitively use the term “random composite”, though a considered picture can be deterministically described, i.e., after the corresponding image and spectral analysis, one can say where and what is located in the picture. Randomness may show itself in the external control parameter, such as the temperature of the technological process, e.g., stir/sand casting [15,16]. The classification problem of the obtained materials and their dependence on the control parameters can be considered in the framework of statistics.

Simple observations and measurements may give a restricted set of geometrical parameters, such as the concentration *f* and two-point correlation functions of phases. Using these geometrical parameters for dispersed composites may lead to analytical formulas for the effective constants at most up to $O(f^{3})$. Higher-order formulas can be derived by structural sums [17,18]. Recent progress in artificial intelligence (AI), particularly in machine learning (ML), has opened new opportunities for analyzing and classifying microstructural patterns beyond traditional statistical approaches. ML enables automated recognition of geometrical regularities and correlations that remain hidden within large sets of analytical descriptors or digitized images. In the context of composites, this approach supports not only prediction of effective properties but also quantitative comparison of randomization protocols and structural irregularities.

Machine learning (ML) and artificial intelligence (AI) offer powerful tools for analyzing complex, random, and dispersed composites. However, many existing studies rely on purely numerical or empirical implementations, often limited to finite element simulations of selected datasets without a clear theoretical linkage to homogenization or analytical modeling. As emphasized in [19], such approaches may yield inconsistent or physically unverified results. In contrast, the present work employs ML within a rigorous analytical framework, ensuring that each feature and prediction step remains grounded in the constructive theory of effective properties.

ML may be effectively applied to special types of composites. For instance, laminates under varied layer orientations during tensile tests were successfully investigated in [20]. Data characterizing the mechanical load behavior were obtained by using twelve composite laminates with different layer orientations. It is important that the special software assigned to composite laminates was used. Compressive strength prediction of steel fiber-reinforced concrete was discussed in [21]. One can find other results of this type concerning particular problems and other algorithms in [22].

To effectively characterize dispersed composites in materials science, datasets must include both the spatial distribution of inclusions, ideally represented through digitized images, and the mechanical properties of the constituent phases depicted therein. These tasks of image analysis were addressed in [23,24]. For our purposes, we assume access to a geometrical dataset detailing the inclusions. Specifically, we consider unidirectional fiber-reinforced elastic composites. A representative cross-section of such a composite is illustrated in Figure 1, where the inclusion coordinates A and their associated mechanical properties M are known. An example of the dataset may be the plane coordinates of centers $A=\{a_{1},a_{2},\ldots ,a_{N}\}$ of equal non-overlapping circular inclusions and the pairs of elastic moduli

(1) $$M=\{\left(\mu ,k\right),\left(\mu_{1},k_{1}\right),\left(\mu_{2},k_{2}\right),\ldots ,\left(\mu_{N},k_{N}\right)\},$$

where μ and *k* denote the elastic shear and bulk moduli of host; $\mu_{m}$ and $k_{m}$ the elastic moduli of *m*th inclusion. The concentration of different phases is a key parameter. In the case of a two-phase composite, we have $M=\{\left(\mu ,k\right),\left(\mu_{1},k_{1}\right)\}$ and the total concentration of inclusions *f*.

Figure 1 illustrates the dataset {A,M}, comprising the plane coordinates of inclusions and their corresponding mechanical properties, arranged in a specific configuration. It is worth noting that a plane section of macroscopically isotropic 3D dispersed composites can adequately represent the considered 3D composites [25].

If the analysis is limited to a particular composite represented by a single picture, it suffices to input the given dataset into a standard finite element method package to compute the effective properties. Such computations are feasible when the number of inclusions per representative volume element (RVE) does not exceed approximately 100. However, for configurations involving around 1000 inclusions, direct computation becomes practically intractable. In such a case, one may partition the RVE into smaller subdomains to facilitate numerical analysis of local fields, but not the effective constants [26].

Simulations involving uniform, non-overlapping distributions with fixed mechanical properties have been conducted in prior studies [27,28], where the number of inclusions per RVE was typically on the order of 30 [14]. The term “random” has been widely used in these and subsequent publications, often without a rigorous definition of the underlying stochastic model. In reality, an infinite variety of probabilistic distributions can be employed to capture different modes of clustering and interaction arising from chemical, biological, or mechanical processes within composite materials. The first results on using ML, the Naive Bayes classifier, for various types of 2D structures were obtained in [13].

In the present paper, advanced machine learning algorithms are employed, including ensemble (bagging) tree-based models and dimensionality-reduction techniques such as PCA and *t*-SNE, to analyze structural sums as geometric features of composites. In the present study, machine learning is not treated as a “black box” numerical tool, but rather as an analytical extension of the constructive homogenization framework developed in Parts I and II. The goal is to integrate symbolic descriptors, structural sums, with data-driven algorithms capable of capturing nonlinear dependencies between geometry and macroscopic behavior.

Specifically, supervised models (ensemble decision-tree regressors and classifiers) are applied to distinguish generation protocols (*R*, *T*, *P*). Unsupervised techniques, such as Principal Component Analysis (PCA) and *t*-distributed Stochastic Neighbor Embedding (*t*-SNE), are employed to visualize high-dimensional geometric relationships and verify the separability of microstructural families.

Each sample is represented by a feature vector derived from structural sums, complex-valued quantities encoding spatial distributions. Their real, imaginary, and modulus components form a multidimensional space analogous to latent representations in modern AI. This allows the combination of physics-informed and statistical learning approaches, where analytical relations constrain the search for correlations detected by the model.

In the context of materials science, we develop a computationally effective strategy to classify very similar dispersed composites that are not distinguishable by direct observations and other methods, such as using the correlation function or pure numerical methods (FEM). Our method can be applied to the investigation of particle interactions and clustering analysis of dispersed composites by their microstructure.

## 2. Schwarz’s Method and Decomposition Theorem

The generalized alternating Schwarz method can be interpreted as an infinite sequence of mutual interactions among inclusions within the boundary value problem formulated for a composite material [17]. The classical Schwarz approach for overlapping domains is typically associated with decomposition techniques widely applied in purely numerical computations. In contrast, the generalized alternating method for non-overlapping inclusions proves advantageous for the symbolic-numerical strategies discussed in this work. Implementing both explicit and implicit schemes leads to new approximate analytical expressions for the effective properties of dispersed composites. Furthermore, the accuracy of these formulas is quantified with respect to concentration and contrast parameters [29]. It is worth noting that the first iteration of this procedure coincides with Maxwell’s well-known self-consistent method [30].

Integral equations in a Banach space associated with Schwarz’s method were first formulated in [31] and later refined in [29] along with related works. In what follows, we present these equations in a general operator form, omitting certain technical details discussed in [29,32]. Let $u_{0}$ denote the prescribed external potential, and $u_{k}$ the unknown potential within the *k*th inclusion $D_{k}$ (k=1,2,…,N). In many physical settings, the potentials $u_{k}$ and $u_{m}$ are connected through a linear operator equation defined in a Banach space

(2) $$u_{k}=\varrho_{k}G_{k}u_{k}+\sum_{m\neq k}\varrho_{m}G_{m}u_{m}+u_{0},\;in\;D_{k},\;k=1,2,\ldots ,N,$$

where $G_{m}u_{m}$ denotes the field in the domain $D_{k}$ induced by the inclusion $D_{m}$. The term $G_{k}u_{k}$ produces the self-induced field.

The physical contrast parameter $\varrho_{m}$ is a multiplier on the bounded operators $G_{m}$. The operator $G_{m}$ is determined by the impact of the local field in the *m*th inclusion onto the field in the *k*th one (m≠k). The operator $G_{k}u_{k}$ has the same form as in the integral equation for a potential $U_{k}$ in a single inclusion $D_{k}$

(3) $$U_{k}=\varrho_{k}G_{k}U_{k}+U_{0},\;in\;D_{k}.$$

Each operator $G_{m}$ implicitly depends on the concentration of inclusions *f*. After a constructive homogenization procedure, the dependence on *f* can become explicit. Moreover, the operator $G_{m}$ does not depend on the material constants. This concerns heat conduction, elastic stress and strain fields, and other processes; see Universality in Mathematical Modeling, Table [19] (Chapter 8). Application of the successive approximation method to Equation (2) leads to a power series in the variables $\varrho_{k}$ (k=1,2,…,N) with the pure geometrical coefficients consisting of operator compositions $G_{m_{1}}\circ \cdots \circ G_{m_{K}}u_{0}$. The explicit form of the operator $G_{m}$ and convergence of this series were discussed in [17,18,29], and works cited therein.

After averaging the field over a representative cell [6], we arrive at the effective constants of dispersed composites. The series for the local fields is transformed into a series for the effective constants. Due to the linearity of $G_{m}$, it is a power series in the physical variables $\varrho_{k}$ and the geometrical coefficients consisting of the integrals on $G_{m_{1}}\circ \cdots \circ G_{m_{K}}u_{0}$. Therefore, the effective constants can be decomposed into a linear combination of pure physical and geometrical parameters of composites. This leads to the following fundamental theoretical result formulated for the effective permittivity tensor.

**Decomposition Theorem** [32] (p. 25). The effective property tensor can be expressed as a linear combination of purely geometrical parameters of the inclusions, with coefficients determined by the local physical constants.

## 3. Structural Sums

The decomposition theorem discussed in the previous section was constructively realized for 2D conductive and elastic composites in Part I and Part II, respectively. The geometrical part of the derived analytical formulas for a composite with circular inclusions was expressed in terms of structural sums. Below, we summarize the general analytical formulas for the sums and examples of their simulations.

### 3.1. General Formulas for Structural Sums

Following the principles of homogenization discussed in Parts I and II, we consider the Representative Volume Element (RVE) formed by two fundamental translation vectors $\omega_{1}$ and $\omega_{2}$. For definiteness, the unit square cell is considered with the vectors expressed through complex numbers $\omega_{1}=1$ and $\omega_{2}=i$.

First, introduce the lattice sum. Let $m_{1}$, $m_{2}$ run over the integer numbers Z. The Eisenstein–Rayleigh lattice sums are introduced by means of the series

(4) $$S_{m}=\underset{m_{1},m_{2}\in Z}{\sum^{\prime }}\frac{1}{{\left(m_{1}\omega_{1}+m_{2}\omega_{2}\right)}^{m}}=\underset{m_{1},m_{2}\in Z}{\sum^{\prime }}\frac{1}{{\left(m_{1}+im_{2}\right)}^{m}},\;m=2,3,\ldots ,$$

where one term $m_{1}=m_{2}=0$ is skipped in the summation. The double series (4) is conditionally convergent for m=2. Its value depends on the order of summation. It was established that $S_{2}=\pi$ for antiplane strain in Part I, and $S_{2}=0$ for plane strain in Part II. The explanation of this seemingly strange fact is based on the conditional convergence of $S_{2}$ first discussed by Rayleigh [33], see also [17,18]. In this paper, we deal with elastic composites; hence, we take $S_{2}=0$.

Eisenstein’s series are defined by the functional series [34]

(5) $$E_{m}\left(z\right)=\sum_{m_{1},m_{2}\in Z}\frac{1}{{\left(z-m_{1}-im_{2}\right)}^{m}}.$$

The elliptic Weierstrass function ℘(z) and the Eisenstein functions are related by identities [34]

(6) $$E_{2}\left(z\right)=℘\left(z\right)+S_{2},\;E_{l}\left(z\right)=\frac{{\left(-1\right)}^{l}}{(l-1)!}\frac{d^{l-2}℘\left(z\right)}{dz^{l-2}},\;l=3,4,\ldots .$$

The Eisenstein–Natanzon–Filshtinsky lattice sums are defined as [18,35,36]

(7) $$S_{m}^{\left(j\right)}=\underset{m_{1},m_{2}}{\sum^{\prime }}\frac{{\left(\bar{m_{1}\omega_{1}+m_{2}\omega_{2}}\right)}^{j}}{{\left(m_{1}\omega_{1}+m_{2}\omega_{2}\right)}^{m}}=\underset{m_{1},m_{2}}{\sum^{\prime }}\frac{{\left(m_{1}-im_{2}\right)}^{j}}{{\left(m_{1}+im_{2}\right)}^{m}},\;m=2,3,\ldots ,\;j=0,1\ldots \;\left(m\geq j+2\right).$$

The series (7) is conditionally convergent for m=j+2. The local stresses and strains for the normalized hexagonal RVE were represented by a power series in *f* [18]. It was established that in this case, we must apply the Eisenstein summation [34] for the coefficients of this series. In particular, this yields [37] $S_{3}^{\left(1\right)}=\frac{\Gamma^{8}\left(1/4\right)}{384\pi^{3}}+\frac{\pi }{2}$ for square RVE and $S_{3}^{\left(1\right)}=\frac{\pi }{2}$ for hexagonal RVE (Γ here denotes the Euler Gamma function). At the same time, a rational representation for the effective elastic constants was derived in [37] and Part II. It turns out that in this case, we must use the symmetric (Maxwell) summation method for which $S_{3}^{\left(1\right)}=\frac{\Gamma^{8}\left(1/4\right)}{384\pi^{3}}\approx 2.5077$ for square RVE and $S_{3}^{\left(1\right)}=0$ for hexagonal RVE. We considered only hexagonal and square cells, but suggest that for a general normalized cell, the difference between Eisenstein’s value $S_{3}^{\left(1\right)}$ and Maxwell’s value $S_{3}^{\left(1\right)}$ holds $\frac{\pi }{2}$.

Let us introduce the functions [18]

(8) $$E_{3}^{\left(1\right)}\left(z\right)=\sum_{m_{1},m_{2}\in Z}\frac{\bar{z+m_{1}\omega_{1}+m_{2}\omega_{2}}}{{\left(z+m_{1}\omega_{1}+m_{2}\omega_{2}\right)}^{3}},$$

which can be determined using the series (7) as follows

(9) $$E_{m}^{\left(j\right)}\left(z\right)=\frac{\bar{z}}{z^{m}}+\sum_{p=0}^{\infty }{\left(-1\right)}^{p}\frac{(m+p-1)!}{p!(m-1)!}\left[\sum_{k=0}^{j}\frac{j!}{k!(j-k)!}{\bar{z}}^{j-k}S_{m+p}^{\left(k\right)}\right]z^{p},$$

where m+p≥j+2.

The most comprehensive computationally effective formulas for the Eisenstein and Natanzon–Filshtinsky series and functions are selected in [17] (Section B.3.3), [18] (Sections A.2 and A.3), [36] (Appendix 2), and [32] (Appendix A).

We are now ready to introduce the most important object of the present paper, the structural sums. Let C denote the complex conjugation operator, *p* be a natural number, *j* can take the values 0 and 1, s=1,2,…,p, $k_{s}=1,2,\ldots ,N$, $\alpha =\sum_{s=1}^{p}\left(n_{s}-j_{s}\right)$, $n_{s}=2,3,\ldots$. Following [18] (Chapter 4), we introduce the elastic structural sums of multi-order $n=(n_{1},\ldots ,n_{p})$

(10) $$e_{n_{1},\ldots ,n_{p}}^{\left(j_{1},\ldots ,j_{p}\right)\left(l_{1},\ldots ,l_{p}\right)}=\frac{1}{N^{\frac{\alpha }{2}+1}}\sum_{k_{0},k_{1},\ldots ,k_{p}}\prod_{s=1}^{p}C^{l_{s}}E_{n_{s}}^{\left(j_{s}\right)}\left(a_{k_{s-1}}-a_{k_{s}}\right).$$

Here, it is assumed for shortness that $E_{n}^{\left(j\right)}\left(a_{l}-a_{s}\right):=S_{n}^{\left(j\right)}$ for the matching centers $a_{l}=a_{s}$. The general structural sums (10) are used for 2D elastic problems in Part II. The following sums written here for even *q* were used for antiplane strain (conductivity) in Part I

(11) $$e_{n_{1},\ldots ,n_{p}}:=e_{n_{1},\ldots ,n_{p}}^{\left(0,\ldots ,0)(0,1,\ldots ,0,1\right)}.$$

An effective computational algorithm to compute the structural sums is presented in [18] (Chapter 2). It was proven that the complexity of the algorithm in the number of inclusions per a periodicity cell, *N*, theoretically does not exceed $N^{2}$ and practically slightly exceeds $N^{1}$. This algorithm ultimately resolves the computational task by applying high-order correlation functions to the effective properties of composites, theoretically discussed in [38,39].

### 3.2. Analysis of Structural Sums

Decomposition theorem for a 2D elastic composite with equal circular inclusions represented by the unit square RVE yields a representation of the effective elastic tensor in the form of a power series in the concentration *f*. Here, for a two-phase composite, the set (1) is reduced to four parameters set $M=\{\left(\mu ,k\right),\left(\mu_{1},k_{1}\right)\}$. The concentration holds $f=N\pi r^{2}$, where *N* is the number of non-overlapping disks in the unit square, *r* is their radius.

We define a classification class for a composite represented by a set of centers A through the set of all the structural sums (10) calculated for A. It was established in Part II that the coefficient $c_{k}$ on $f^{k+1}$ for the effective elastic constants is a linear combination of the structural sums $e_{n_{1},\ldots ,n_{p}}^{\left(j_{1},\ldots ,j_{p}\right)\left(l_{1},\ldots ,l_{p}\right)}$ with $n_{1}+\ldots +n_{p}=2k$. Therefore, an infinite set of structural sums denoted by $E_{\infty }\left(N\right)$ can be assigned to any composite besides four constants M. For simplicity of further analysis, we fix the material constants M and study only the geometrical parameters $E_{\infty }\left(N\right)$ calculated for the set M.

The set $E_{\infty }\left(N\right)$ is infinite and may be truncated by the concentration order. We use the results of Part II and calculate the finite set of structural sums for A

(12) $$\begin{array}{c} E_{14}\left(N\right)=\left\{e_{3}^{\left(1)(1\right)},e_{4}^{\left(0)(1\right)},e_{22}^{\left(00)(10\right)},e_{22}^{\left(00)(11\right)},e_{33}^{\left(11)(10\right)},e_{33}^{\left(00)(10\right)},e_{43}^{\left(01)(10\right)},e_{34}^{\left(10)(10\right)},e_{44}^{\left(11)(10\right)},\right.\\ \left.e_{333}^{\left(111)(101\right)},e_{223}^{\left(001)(001\right)},e_{223}^{\left(001)(110\right)},e_{223}^{\left(001)(101\right)},e_{232}^{\left(010)(010\right)}\right\}\equiv \left\{E_{1},E_{2},\ldots ,E_{14}\right\}. \end{array}$$

According to Part II, we need these 14 structural sums to determine the effective constants up to $O(f^{5})$. Let us present the expansions of the normalized effective shear and bulk moduli for macroscopically isotropic composites in the form

(13) $$\begin{array}{c} \frac{\mu_{e}}{\mu }=1+c_{1}f+c_{2}f^{2}+c_{3}f^{3}+c_{4}f^{4}+O\left(f^{5}\right),\\ \frac{k_{e}}{k}=1+d_{1}f+d_{2}f^{2}+d_{3}f^{3}+d_{4}f^{4}+O\left(f^{5}\right). \end{array}$$

The coefficients $c_{1}$, $c_{2}$, $d_{1}$, $d_{2}$ coincide with the coefficients of the expansions of Hashin–Shtrikman universal lower bounds in the case $\mu_{1}>\mu$ and $k_{1}>k$ and do not depend on the location of inclusions. The coefficients $c_{3}$, $c_{4}$, $d_{3}$, $d_{4}$ are linear combinations of the structural sums (12) explicitly written in Part II, hence, depend on the location of inclusions.

The numerical dataset models a microstructure composed of N=100 circular inclusions distributed in a doubly periodic unit cell represented by the two-dimensional plane torus. Four concentration levels of inclusions were considered, namely, f=0.1,0.2,0.3, and 0.4. Three distinct generative procedures R,T, and *P* described in Part II are used. Every procedure is applied 100 times to four concentrations F={0.1,0.2,0.3,0.4} and next averaged over 100 experiments. Hence, we perform 100·3·4=1200 single computations, and have 12 groups of vectors (12) after averaging. Although all the procedures theoretically correspond to the uniform distribution of non-overlapping disks and, moreover, follow the random sequential adsorption protocol (RSA) [40], they differ in the way center positions are sampled and adjusted. The extent description of the protocols R,T, and *P* can be found in Section 6.2 of Part II.

Variant *R* corresponds to the classical random sequential adsorption scheme in which disks are placed sequentially at random positions and each new candidate is accepted only if it does not overlap with any previously placed disk; otherwise, it is rejected and resampled. Variant *T* starts with a set of randomly drawn points that are subsequently regularized through a cleaning procedure: whenever two points are too close, they are replaced by their geometric midpoint, and the process is repeated iteratively until all disks satisfy a prescribed minimum separation distance. This approach yields random yet more uniformly distributed arrangements. Variant *P* begins with an overlapping configuration and resolves overlaps through an iterative settling process, in which disks are displaced away from each other along their connecting line until no overlaps remain. This procedure resembles the physical deposition of grains or particles and allows the efficient generation of denser packings while preserving randomness.

The resulting collection is subjected to ML analysis in the next section to investigate the impact of generation protocols and concentration on the descriptors and, ultimately, on predictive modeling of macroscopic material properties.

## 4. ML Methods

We now proceed to apply ML to the considered above composites. The input for classification of dispersed composites is the family of 14-dimensional vectors (12). Traditionally, the ML approach has its own set of designations that differ from those used in academia. For convenience, we select the ML designations at the end of the paper.

### 4.1. Feature Construction from Structural Sums

The dataset comprises the following: a categorical label *type* ∈{R,T,P}; a value of concentration f∈F={0.1,0.2,0.3,0.4}; and multiple columns of structural sums (12) in complex format a+bi. For each structural sum z=a+bi we extract four elemental features:

Rez=a,Imz=b,|z|=a2+b2,argz=arctgba,ifa>0,arctgba+π,ifa<0.

We compute family-level aggregates over structural sum index families. Each family corresponds to a group of structural sums that share the same order and thus represent a common geometric hierarchy. For example, a family such as $\left\{E_{1},E_{2},E_{3}\right\}$ includes all structural sums but differing in index combinations. Within every family, descriptive statistics are calculated, including the mean and standard deviation of the real parts Rez, magnitudes |z|, and arguments argz. These aggregates summarize intra-family variability and capture geometric trends common across related sums. In addition, derived descriptors—such as the ratio of real to imaginary parts and normalized amplitude differences—are introduced to measure deviations from geometric symmetry.

The number of ML features is reported in this subsection. The considered hierarchical representation yields approximately ∼130 features, improving both the interpretability and stability of the subsequent ML analysis. All features are standardized via *z*–score normalization (zero mean, unit variance). Principal Component Analysis (PCA) reveals about 36 effectively independent components, reflecting collinearities among raw features.

#### Dataset Generation and Implementation Details

The dataset used in this study consists of 1200 composite configurations. For each generation protocol (R,T,P) and each concentration level f∈{0.1,0.2,0.3,0.4}, we generated 100 independent realizations containing N=100 non–overlapping inclusions per periodic cell. The corresponding 14 structural sums listed in (12) were exported in complex format and combined into a tabular dataset together with the class label (R/T/P) and concentration *f*.

All data processing and machine-learning analyses were performed in MATLAB R2023b. The complex values of structural sums were parsed from their textual representation (“a+b⁢I”) into MATLAB complex numbers. For each structural sum *z*, four numerical descriptors were computed: real part Rez, imaginary part Imz, magnitude |z|, and argument argz. Additionally, simple family-level aggregates were incorporated (e.g., grouping sums indexed as “22”, “33”, “44”), resulting in approximately 130 raw features. All features were standardized using MATLAB’s zscore function.

The R/T/P classification task was performed using a bagging ensemble of decision trees (fitcensemble with default templateTree learners). Model performance was evaluated using stratified 5-fold cross-validation executed via cvpartition and crossval, ensuring balanced representation of all three classes in every fold. The mean cross-validated classification accuracy was ACC=0.7125. To ensure full reproducibility, we specify the exact configuration of the ensemble model. The classifier was implemented using MATLAB’s fitcensemble function with the ‘Bag’ method and the default templateTree base learner. This corresponds to an ensemble of 200 decision trees trained on bootstrap-resampled datasets, with each tree grown without predetermined depth constraints (unpruned CART trees). At each split, all available predictors were considered (NumVariablesToSample = ‘all’), and no additional feature-selection or dimensionality reduction step was applied prior to training. This configuration yields a low-bias, high-variance base learner whose variance is reduced through averaging across trees, consistent with classical bagging theory.

The number of trees (200) and the unconstrained depth were verified to be sufficient by monitoring the out-of-bag error curve, which plateaued well before reaching the full ensemble, indicating that variance reduction had stabilised. Increasing the number of trees to 300 or limiting tree depth did not produce statistically meaningful changes in cross-validated accuracy (variations below ±0.01), confirming that the reported ACC = 0.7125 is robust with respect to ensemble size and model capacity. No regularisation or pruning was applied, as deep trees are known to be optimal base learners for bagging. Dimensionality reduction was applied for visualization and interpretability. Principal Component Analysis (PCA) was performed using MATLAB’s pca function; the first two principal components explained approximately 47.6% and 11.0% of the variance, respectively, and already revealed clear separation between R, T, and P samples. A nonlinear embedding was additionally obtained using tsne, confirming local cluster separability.

Feature relevance was quantified using permutation importance computed via out-of-bag predictor perturbations (oobPermutedPredictorImportance). Higher-order magnitudes |z| and real parts Rez consistently ranked as the most informative descriptors, in agreement with the theoretical expectation that higher-order structural sums encode finer geometric information.

To illustrate discriminability at low dimensionality, all two-feature combinations were evaluated using a *k*–nearest neighbours classifier (k=5, 5–fold CV). This procedure identified several highly separative pairs, enabling intuitive two-dimensional visualizations of class boundaries.

The PCA was used exclusively for visualization and dimensionality reduction diagnostics, not for model training. The observation that approximately 36 principal components capture most of the variance indicates internal redundancy within the engineered feature set, but the classifiers always operated on the full set of engineered features. Model-agnostic interpretability techniques such as permutation importance are therefore the primary tool used here to quantify feature relevance in the full engineered space; methods such as SHAP, while applicable in principle, were not required for the present analytical objectives of the study.

For transparency, a complete list of all feature definitions is provided in the Supplementary Materials. A structured summary of all engineered feature groups used for model training is provided in Table S1 in the Supplementary Materials.

**Figure 2:**
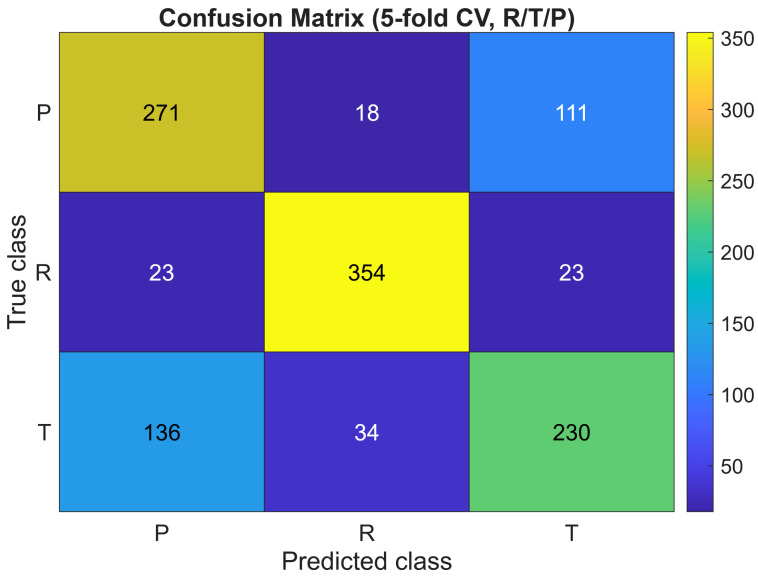
Confusion matrix for *R/T/P* classification obtained from 5-fold cross-validation predictions of the bagging ensemble trained on the full engineered feature set (130 features). The mean cross-validated accuracy is ACC = 0.7125.

To assess the reliability of the classifier beyond aggregated metrics, we also examined patterns in the misclassified samples and evaluated the robustness of the model under perturbations. As visible in the confusion matrix (Figure 2), the majority of errors occur between the *R* and *T* classes, which is consistent with their partial geometric overlap observed in both the PCA and t–SNE embeddings. The *P* class remains the most distinct owing to its pronounced regularity. A misclassification inspection reveals that mislabelled cases tend to have intermediate values of higher-order magnitudes |z| and phase-variability descriptors, confirming that errors arise from genuinely ambiguous geometries rather than numerical instability.

To test sensitivity to noise, we added independent Gaussian perturbations to all features (σ=5% of the empirical standard deviation) and retrained the classifier. The resulting cross-validated accuracy varied by less than ±0.01, indicating strong numerical stability. Doubling the sampling density (by regenerating the dataset with 200 realizations per class and concentration) produced the same qualitative separation between *R*, *T*, and *P* and changed the accuracy by less than ±0.015. Furthermore, the classifier was evaluated on modified datasets in which the inclusion radii were varied by ±5% while preserving non-overlap; no statistically significant degradation of performance was observed. These tests collectively indicate that the model is robust to perturbations in sampling density, numerical noise, and moderate geometric variations in location of inclusions, and that misclassified cases reflect intrinsic transitional microstructures rather than model instability.

### 4.2. Accuracy ACC

We use a bagging ensemble of decision trees for the classification (R/T/P), cross-validation (5-fold CV),

(14) $$\mathrm{Accuracy}\;\left(\mathrm{ACC}\right)=\frac{\#\;\mathrm{correct}\;\mathrm{predictions}}{n},$$

where *n* denotes the total number of test samples, and #(correctpredictions) is the number of samples whose predicted labels match the true class labels.

Accuracy quantifies the proportion of correctly classified samples among all observations. ACC = 1 corresponds to perfect classification, whereas a value close to the random baseline (approximately 0.33 for three classes R/T/P) indicates low discriminative capability. In this study, ACC measures the ensemble classifier’s ability to correctly identify the composite type (R,T, or *P*) based on geometric descriptors derived from structural sums.

The classification accuracy ACC thus provides a concise and reliable value for verifying that the structural–sum-based features preserve sufficient geometric information to distinguish between microstructure generation protocols. This metric is consistently used in the subsequent sections to evaluate the performance of the ensemble models. The confusion matrix presented in Figure 2 corresponds directly to these 5-fold cross–validation predictions, ensuring full consistency between the reported accuracy ACC = 0.7125 and the sample–wise classification outcomes.

### 4.3. Visualization and Feature Interpretation

Principal Component Analysis (PCA) provides a linear two-dimensional projection that highlights the global structure. *t*–distributed Stochastic Neighbor Embedding (*t*–SNE) gives a nonlinear embedding preserving local neighborhoods by minimizing the Kullback–Leibler divergence

(15) $$\mathrm{KL}\left(P∥Q\right)=\sum_{i\neq j}p_{ij}\log\frac{p_{ij}}{q_{ij}},$$

with $p_{ij}$ and $q_{ij}$ denoting pairwise similarities in the input and embedded spaces, respectively. Permutation importance quantifies feature relevance as the drop in ACC after randomly permuting a given feature. Best feature pairs are identified by enumerating all two-dimensional pairs, training *k*–nearest neighbors (*k*–NN with k=5) on each pair, and selecting the highest 5-fold CV ACC. To make the methodology explicit, the selection of “best feature pairs” proceeds as follows. From the standardized feature matrix containing approximately 130 engineered descriptors derived from the fourteen structural sums in Equation (12), we enumerate all unordered pairs $\left(x_{i},x_{j}\right)$. For each candidate pair, we train a *k*-nearest neighbours classifier (fitcknn, k=5, Euclidean distance) using only the two-dimensional input $\left(x_{i},x_{j}\right)$ and evaluate its performance via stratified 5-fold cross-validation.

The mean cross-validated accuracy serves as the scoring criterion for each feature pair, and the highest-scoring pairs are retained for subsequent visualization.

This procedure is intended purely as an interpretable, model-agnostic probe of low-dimensional separability, rather than as an alternative classifier for the main R/T/P task. Higher-dimensional feature subsets (k>2) could also be selected, yet two-dimensional projections uniquely enable geometric visualization of class separation, which is essential for interpreting how individual structural–sum descriptors encode microstructural differences.

In parallel, global feature relevance in the full 130-dimensional space is quantified via permutation importance for the bagging-tree ensemble (oobPermutedPredictorImportance). The resulting importance profiles consistently show that higher-order magnitudes |z| and real-part components Rez dominate predictive performance, while lower-order aggregates contribute less. These findings align with the theoretical argument that higher-order structural sums encode the finest geometric information.

SHAP-style analyses could be performed, but for tree ensembles the permutation-based approach already provides stable, model-agnostic, and easily interpretable feature rankings, which suffices for the analytical objectives of this study.

### 4.4. Degree of Irregularity

Let ${∥c∥}_{2}=\sqrt{\sum_{i=1}^{n}c_{i}^{2}}$ denote the Euclidean norm $\ell_{2}$. Consider the following three standardized components:

- **High-order energy** $z_{E}$ is the mean of |z| over higher-index families (captures intensity of higher-order content),
- **Phase chaos** $z_{\phi }$ denotes the standard deviation of argz over selected families (captures angular variability),
- **Im/Re asymmetry** $z_{A}$ is the ratio of the $\ell_{2}$ norms of the imaginary and real components across selected structural-sum families,
  (16) zA=∥Imz∥2∥Rez∥2.

The value (16) quantifies the imbalance between the amplitudes of the imaginary and real parts in the complex plane and, therefore, reflects deviations from geometric symmetry in the spatial arrangement of inclusions. To give a simple example, consider the case of one-element vector $z=\{e_{2}\}$, where the structural sum is defined by (11) with p=1 and $n_{1}=2$. Write it for clarity in the explicit form

(17) $$e_{2}=\frac{1}{N^{2}}\sum_{k,m=1}^{N}E_{2}\left(a_{k}-a_{m}\right),$$

where $E_{2}\left(z\right)$ is defined by (6) and can be calculated by (5). Here, it is assumed for shortness that $E_{2}\left(a_{k}-a_{m}\right)=\pi$ for k≠m. Then, the value zA=Ime2Ree2 determines the degree of macroscopical isotropy, since $e_{2}=\pi$ for ideally isotropic composites [23]. In addition to this, the second equation must hold $e_{3}^{\left(1)(1\right)}=\frac{\pi }{2}$ for plane strain [18]. It is worth noting that the Eisenstein summation is applied in this example.

We define the irregularity index $\eta^{*}$ as a linear combination of the above three values rescaled to [0,1]

(18) $$\eta^{*}=0.5\;z_{E}+0.3\;z_{\phi }+0.2\;z_{A}.$$

All three components are made dimensionless and comparable prior to aggregation: $z_{E}$ is min–max normalized over the selected families of |z|, $z_{\phi }$ is computed as the standard deviation of argz and normalized by π, and $z_{A}$ is squashed to [0,1] via x↦x/(1+x) and then min–max scaled. The weights 0.5,0.3,0.2 in (18) emphasize high–order energy while retaining phase variability and Im/Re asymmetry. We verified that small perturbations of these weights do not affect the conclusions. Maybe other combinations do affect the conclusions. These weights were selected based on empirical variance analysis of the 14 structural-sum families and on their relative discriminative power observed in the R/T/P classification task. Higher-order magnitudes |z| contribute the dominant share of variance (approximately 55%), which justifies assigning the largest weight to $z_{E}$. The phase variability component $z_{\phi }$ shows a moderate but systematic class-separating effect, whereas the Im/Re asymmetry $z_{A}$ exhibits the weakest but still non-negligible sensitivity. Therefore, the triplet of weights (0.5,0.3,0.2) reflects the relative statistical importance of the three standardized components while keeping $\eta^{*}$ stable under small perturbations of these weights. To verify that the coefficients in (18) are not arbitrary, we conducted a robustness study using several alternative weighting schemes, including uniform weights (1/3,1/3,1/3), variance-proportional weights, and entropy-based normalization. In all cases, the resulting values of $\eta^{*}$ were strongly correlated with the baseline definition (r>0.97), and the ranking of samples as well as all class-level trends remaining unchanged. This confirms that the triplet (0.5,0.3,0.2) acts merely as a convenient normalized scaling that reflects the relative statistical contributions of the three components, rather than a sensitive or arbitrary choice that would affect the interpretation of the irregularity index. Higher $\eta^{*}$ indicates greater irregularity of the set of composite microstructures, not an irregularity of a single microstructure. Specifically, it reflects stronger deviations from spatial symmetry, higher angular disorder, and increased imbalance between the real and imaginary components of the structural sum families.

It is worth noting the entropy-like irregularity measure introduced in [13,18], which conceptually relates to the present definition but was derived for different classes of composites. We now extend this notion to 2D elastic composites. Consider the infinite set of structural sums $E_{\infty }\left(N\right)=\left\{E_{1},E_{2},\ldots \right\}$ introduced in the previous section. Let $\left\{E_{1}^{hex},E_{2}^{hex},\ldots \right\}$ be the set of structural sums calculated for the regular normalized hexagonal array of disks with formally fixed N=1. This set consists of the lattice sums (4) and (7) calculated for $\omega_{1}=1$ and $\omega_{2}=i$.

Let for fixed integers *i* and *M* some coefficients from the polynomial approximation of degree *M* (similar to (13)) contain a structural sum $E_{i}\left(N\right)$. Let ℓ(i) denote the minimal index of these coefficients. Introduce the structural irregularity measure similar to [13,18]

(19) $$\chi =\sum_{\begin{array}{c} i=1\\ E_{i}\left(N\right)\neq 0 \end{array}}^{M}f^{\ell (i)}\left|1-\frac{E_{i}^{hex}}{E_{i}\left(N\right)}\right|\ln\left|1-\frac{E_{i}^{hex}}{E_{i}\left(N\right)}\right|.$$

The measure χ holds zero for the regular hexagonal array. It can be considered as the distance in some metric between a structure and the hexagonal array. The measure χ indicates the degree of regularity/irregularity of a single microstructure. Introduction of the measure χ qualitatively resolves the irregularity question of disordered structures traditionally considered in physics, rather quantitatively, as a deviation of random structure from regular lattices [41].

## 5. Results

### 5.1. Quality of Classification and Regression

Using 5-fold cross-validation across the dataset, the R/T/P classifier achieves the accuracy ACC =0.7125, demonstrating that structural sums, transformed into the proposed feature vector, carry sufficient geometric information for classifying the inclusion generation protocols (R,T,P). This confirms the consistency between statistical performance and geometric interpretation of the ML models [13,17,18].

The confusion matrix shown in Figure 2 presents the classification outcomes for the three composite types *R*, *T*, and *P* obtained with the ensemble of decision trees.

The rows correspond to the true classes, while the columns show the predicted ones; cell intensity reflects the number of classified samples. A dominant diagonal pattern confirms a high classification accuracy of the model under 5-fold cross-validation. Off-diagonal values mainly occur between *R* and *T*, indicating partial overlap of transitional geometries, while class *P* remains the most distinct owing to its pronounced respective structural regularity. These results confirm that the ensemble model effectively captures the geometric signal encoded in the structural sums features.

**Figure 3:**
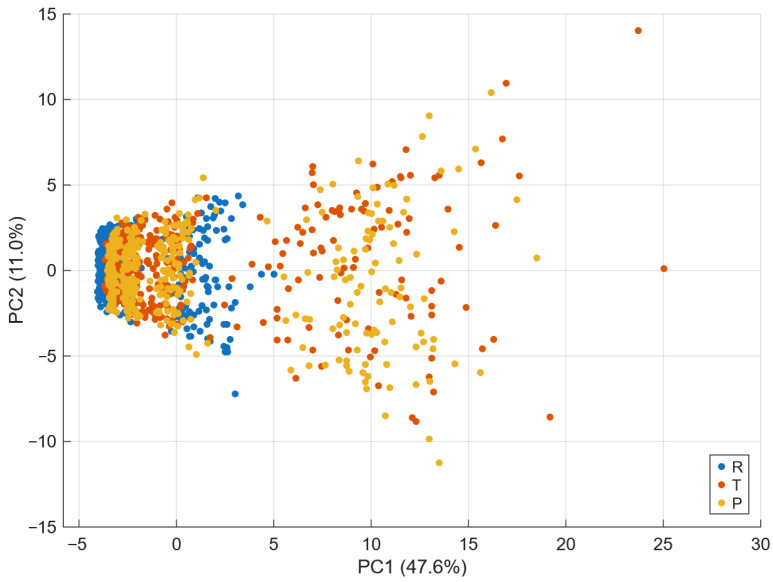
Principal Component Analysis (PCA) two-dimensional projection of the standardized feature set derived from structural sums. Each point represents a composite sample, color-coded by generation protocol (R,T, or *P*). The axes correspond to the first two principal components, PC1 and PC2, which explain 47.6% and 11.0% of the total variance, respectively. The higher explained variance of PC1 indicates that it captures the dominant geometric variability among composites, while PC2 reflects secondary variations related to structural irregularity.

Figure 3 illustrates the two-dimensional Principal Component Analysis (PCA) projection derived from the standardized feature set. The first two components (PC1 and PC2) account for nearly 55% of the total variance, revealing clear spatial separation among the R,T, and *P* composites. Each point represents a sample described by the 14 structural–sum features, projected into the linear space of maximal variance. The visible clustering demonstrates that the constructed feature vector preserves global geometric differences between classes. This confirms that even a linear transformation of the feature space retains sufficient information to distinguish composite morphologies.

**Figure 4:**
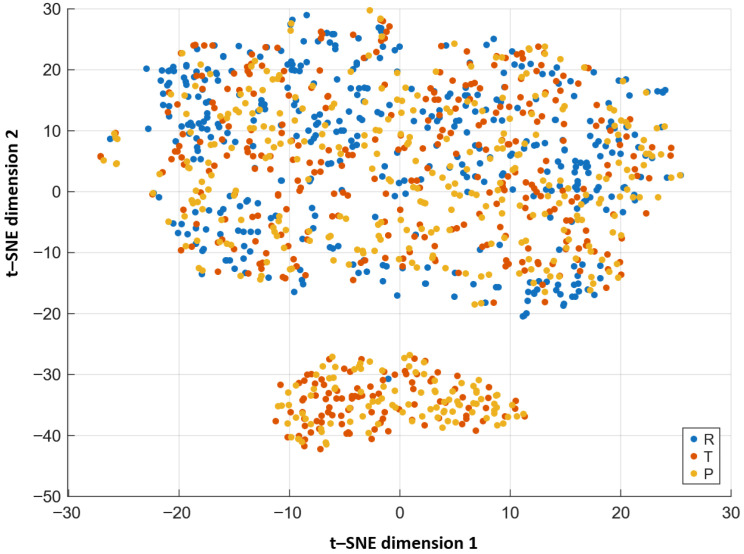
*t–SNE* (*t*-distributed Stochastic Neighbor Embedding) two-dimensional projection. Axes correspond to *t–SNE dimension 1* and *t–SNE dimension 2*. Distinct and compact clusters for classes R,T, and *P* demonstrate that neighboring samples share similar structural characteristics, confirming the nonlinear separability of the microstructures.

The nonlinear projection obtained by *t*–SNE (Figure 4) provides a complementary view to PCA, emphasizing local relations between samples. This method preserves the relative distances in the high-dimensional feature space by minimizing the Kullback–Leibler divergence between the original and embedded data distributions. Distinct and compact clusters for classes R,T, and *P* demonstrate that neighboring samples share similar structural characteristics. The *t*-SNE visualization confirms that the separation observed in the PCA projection also holds in the nonlinear manifold, validating the robustness of the ML descriptors across both global and local scales.

**Figure 5:**
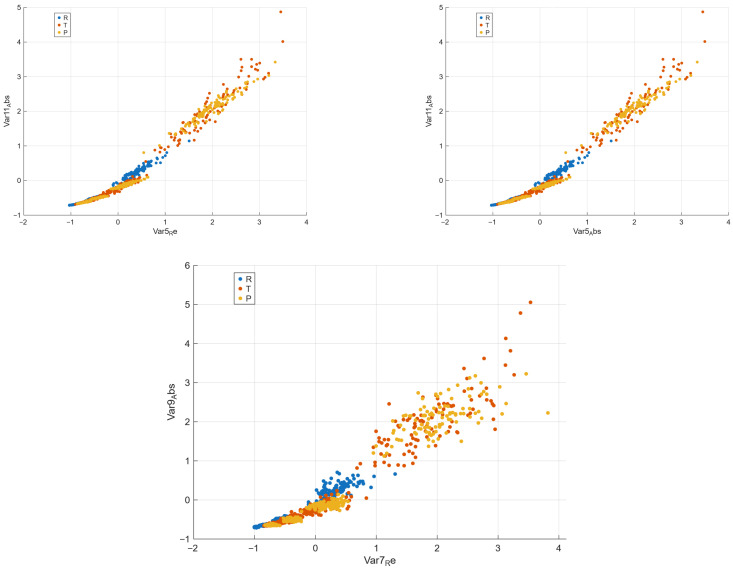
Best two-dimensional feature pairs obtained using the *k*-nearest neighbors classifier (k=5, 5-fold CV). Each scatter plot corresponds to one feature combination yielding the highest classification accuracy (ACC). Colors denote the composite classes (R,T, and *P*). Axes labeled as Var*_i_*Re and Var*_i_*Abs denote the real part and absolute value of the *i*th standardized feature variable, respectively. Each feature variable is derived from a specific structural-sum descriptor and represents a geometric component of the composite microstructure.

Figure 5 shows the top three two-dimensional feature pairs that yield the highest classification accuracy (ACC) using the *k*-nearest neighbors algorithm (k=5) with 5-fold cross-validation. Each scatter plot corresponds to a single feature combination, where colors denote the composite classes (*R*, *T*, and *P*). The left panel generally reflects contrasts in higher-order magnitudes |z|, while the rightmost one involves the real parts Rez of structural sums. Even in two-dimensional projections, the classes are visibly separated, confirming that a limited subset of the 130 derived features already carries strong discriminatory power. This demonstrates that the geometric encoding provided by structural sums effectively captures essential microstructural distinctions between composite types.

**Figure 6:**
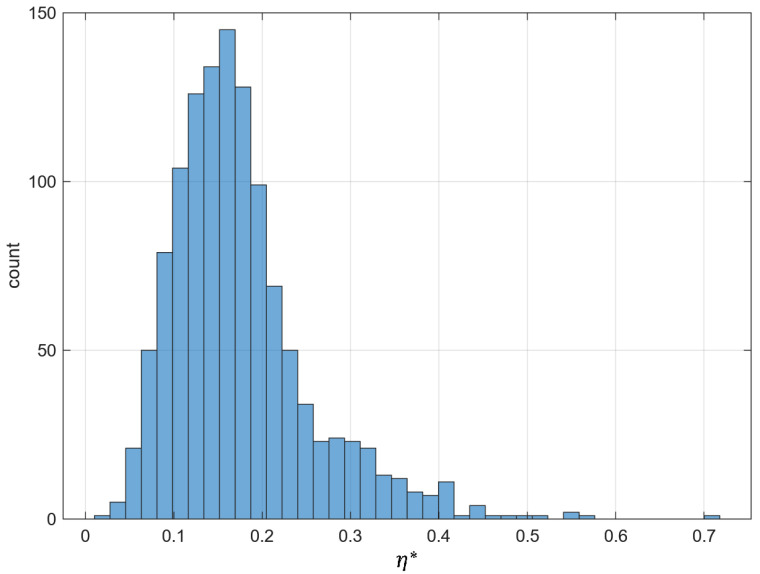
Histogram of the irregularity index $\eta^{*}$ calculated by (17). The vertical axis represents the number of composite samples falling within each $\eta^{*}$ interval (*count*).

Figure 6 displays the histogram of the irregularity index $\eta^{*}$ calculated for the set of all analyzed samples, each corresponding to one composite configuration. The index $\eta^{*}$ was obtained as a weighted sum of standardized components, high-order energy, phase variability, and asymmetry as defined by Equation (14). Lower values of $\eta^{*}$ correspond to highly regular and isotropic structures (typically of type *P*), whereas higher values are associated with more chaotic distributions (mostly of type *R*) that represent partially ordered configurations. The distribution is right-skewed, indicating that strongly irregular microstructures form a smaller subset of the dataset. This visualization provides the basis for further quantitative analysis of how structural irregularity changes with inclusion concentration.

**Figure 7:**
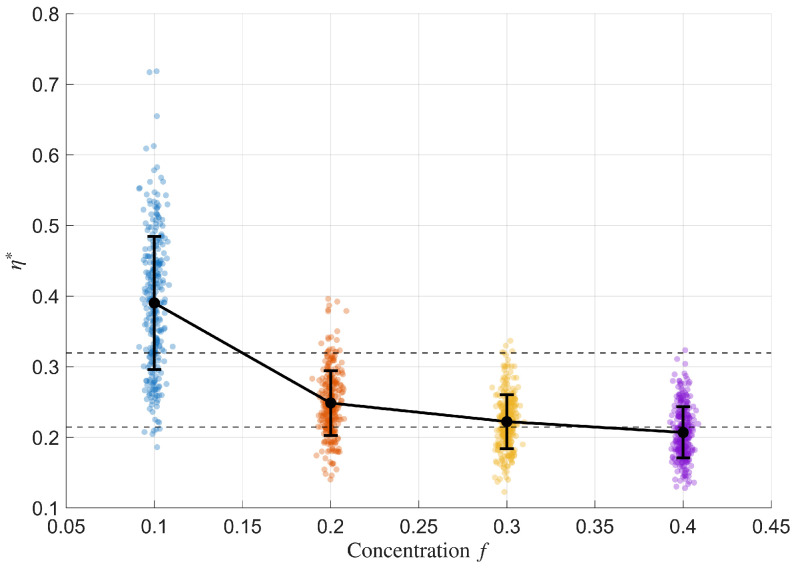
Irregularity index $\eta^{*}$ as a function of concentration *f*. Coloured points denote individual composite samples (1200 realisations); black circles and vertical bars indicate the mean ± one standard deviation at each concentration level. Horizontal dashed lines show the average $\eta^{*}$ values over the low-concentration range f∈[0.1,0.2] and the high-concentration range f∈[0.3,0.4].

Figure 7 displays the dependence of the irregularity index $\eta^{*}$ on the inclusion concentration *f* at the level of individual samples. Each coloured point corresponds to one of the 1200 composite configurations (R,T,P), plotted at its concentration value with a small horizontal jitter to avoid overlap. The black markers and vertical error bars show the mean value and one standard deviation of $\eta^{*}$ for each concentration level f∈{0.1,0.2,0.3,0.4}. The mean irregularity decreases systematically from about $\eta^{*}\approx 0.38$ at f=0.1 to $\eta^{*}\approx 0.24$ at f=0.2, $\eta^{*}\approx 0.22$ at f=0.3, and $\eta^{*}\approx 0.20$ at f=0.4, while the spread of the point cloud also becomes narrower. To emphasise this trend, horizontal dashed lines indicate the average $\eta^{*}$ over the low-concentration range f∈[0.1,0.2] and the high-concentration range f∈[0.3,0.4], equal to $\eta^{*}\approx 0.31$ and $\eta^{*}\approx 0.21$, respectively. This combined scatter-plus-error-bar representation provides a transparent view of both the global decay of $\eta^{*}$ and the sample-level variability within each concentration regime.

Figure 7 therefore substantiates the hypothesis that structural sums encode the progressive transition from random to more ordered composite configurations as the packing fraction increases.

The visual patterns observed in Figure 6 and Figure 7 are further supported by a statistical examination of sample distributions. In the following subsection, we quantify how the differences in the irregularity index $\eta^{*}$ correspond to variations in inclusion concentration *f* and verify that these effects are consistent across all composite classes.

This approach follows the methodology applied in recent analyses of random and clustered composites [13,42,43].

### 5.2. Concentration Analysis (Counts and Classes)

For f∈[0.1,0.2] there are 600 samples with a mean irregularity $\eta^{*}\approx 0.31$ and a balanced class distribution (#R=200, #T=200, #P=200). For f∈[0.3,0.4] there are again 600 samples, but with a smaller mean irregularity $\eta^{*}\approx 0.21$ and the same class counts. Hence, the observed reduction of $\eta^{*}$ between these ranges is driven by the concentration *f* rather than by any change in the class proportions. These quantitative findings are fully consistent with the visual trends in Figure 6 and Figure 7 and confirm that the irregularity index $\eta^{*}$ decreases systematically as the packing fraction increases, indicating a robust geometric stabilization of the microstructures [17,42].

Moreover, the proposed analysis allows for the extension of the results derived in Parts I and II for discrete concentration values f=0.1,0,0.2,0.3,0.4 to continuous f∈(0.1,0.4).

## 6. Discussion and Conclusions

Compared with previous exploratory studies using simple classifiers such as Naive Bayes [13], the present approach extends the methodology toward robust ensemble learning, feature-importance analysis, and interpretable embeddings consistent with the analytical decomposition of effective tensors. Consequently, the ML stage acts as a bridge between the deterministic mathematical model and empirical statistical inference, reinforcing reproducibility and interpretability in the study of random composites. The workflow follows the general paradigm of data-driven materials modeling—dataset construction, feature engineering, training, validation, and interpretation—adapted here to analytical descriptors rather than raw images.

The considered model reaches the accuracy ACC=0.7125 for classification into R/T/P classes under 5-fold cross-validation, which confirms the predictive strength of the constructed feature vector. Visualization methods, namely Principal Component Analysis (PCA) and *t*-distributed Stochastic Neighbor Embedding (*t*–SNE), further confirm the natural separation of the classes. The analysis of the best two-dimensional feature pairs supports the interpretability of the model.

The irregularity index $\eta^{*}$ provides a scalar descriptor that quantifies the set irregularity of composite microstructures. The observed decrease in the mean value of $\eta^{*}$ for higher concentrations (f∈[0.3,0.4]) compared with lower ones (f∈[0.1,0.2]) indicates that denser microstructures tend to be more regular and stabilized at higher packing.

The study demonstrates that structural sums constitute an effective and quantitative descriptor of 2D composite geometry and can be directly used as machine-learning features. Our cycle of papers, based on [17,18,32], essentially extends the previous naive statistical investigations, which were concentrated on the special uniform distribution.

The main advantages and features of the structural sums, the cornerstone of *a*RVE, were partially summarized in [32] (Chapter 4) and given below in the context of the elasticity problems.

- A class of random composites can be directly determined by a set of structural sums without the computation of its effective properties. In the present paper, ML methods is used to reach the goal of classification.
- In the framework of *a*RVE, random clustering composites can be theoretically simulated [13] as well as an observed composite can be investigated [43].
- The macroscopic isotropy can be quickly verified by means of structural sums [40,42] (Equations (3.2)).
- *a*RVE can be applied to any distribution of disks on the plane, not only to the uniformly distributed inclusions tacitly considered in the majority of published works.
- The method does not use expansive, purely numerical computations, such as FEM, infinite systems of equations, and integral equations.
- The method does not use virtually impossible computation of higher-order correlation functions.

Future research will address practical classification challenges, which were preliminarily explored in [23] for the one-dimensional vector (12), simplified to the scalar form $E_{1}=e_{2}$. The present study builds upon the theoretical foundations established in Part II, where stochastic simulations were conducted using three distinct protocols R,T,P, each based on a uniform distribution of identical disks. To illustrate the scheme, we now consider three specific fourth-order approximate expressions from Part II for the normalized effective shear modulus of composites containing hard-particle inclusions:

(20) $$\begin{array}{c} \mu_{e,4}^{R}\left(1,f\right)=1+2f+2.01227f^{2}+10.7687f^{3}+13.806f^{4},\\ \mu_{e,4}^{P}\left(1,f\right)=1+2f+2.0195f^{2}+18.0611f^{3}+20.3269f^{4},\\ \mu_{e,4}^{T}\left(1,f\right)=1+2f+1.94768f^{2}+17.9351f^{3}+18.4812f^{4}. \end{array}$$

In Part II, the structural sums were simulated using the protocols R,T,P. These sums were then averaged within each protocol and substituted into the general formulas derived in Part II, yielding the coefficients of Equation (20). The coefficients in $f^{2}$ exhibit deviations near the value 2, which corresponds to an ideally isotropic composite. The subsequent coefficients in $f^{3}$ and $f^{4}$ encapsulate more nuanced information about the composites, reflecting higher-order correlations. In this paper, we analyze these coefficients in depth by examining their fundamental components, the structural sums. Section 4 demonstrates a strong correspondence between the protocols R,T,P and their respective features, providing theoretical validation for the proposed ML approach.

Looking ahead, practical research will focus on investigating real microstructures. If we need the effective constants of random composites, we follow the strategy developed in Parts I and II, for example, by estimating the constants directly using Formula (20). The results of the present paper can be applied to the classification of a set of microstructures. In addition to the effective constants, the structural-sum feature vectors represent the hidden information on the morphological structure of heterogeneous media.

Let us give a bright example from the study of bacteria. The collective behaviour of bacteria was discussed in [44,45], where the correlation length and correlation time of a bacterial suspension were studied. The onset of collective motion was related to the hydrodynamic interactions versus collisions by studying the effect of the dipole moment. The macroscopically isotropic behavior of bacteria was established in [46,47], with some local oscillations on the mesoscopic level, which can be explained by the distinction between active and passive particles [48]. Taking into account that the effective viscosity of 2D macroscopically isotropic suspensions within the approximation $O(f^{2})$ does not depend on the location of bacteria, we conclude that the results [44,45] are based rather on semi-empirical observations. At the same time, the higher-order structural sums are significantly different for chaotic and collective behavior [46,47]. This clearly demonstrates the advantage of higher-order structural sums.

Come back to material science. Suppose we have several microstructure images produced via the same technological process but governed by three distinct control parameters, denoted by the same letters R,T,P. Instead of relying on theoretical simulations, we can extract data directly from these raw images and apply the ML method outlined in Section 4. The current simulations suggest that it should be possible to classify the composites based on the actual technological parameters R,T,P. If classification fails, it may indicate that these parameters are not fundamentally significant. Naturally, such a negative outcome could also result from excessive deviations in the data.

The extension of ML methods in future research will be applied to multi-phase composites, building on the methods developed in [49,50]. The shape-form impact on classification will also be considered in light of the studies [29,50,51].

## Data Availability

The original contributions presented in the study can be found in the article. Further inquiries can be directed to the corresponding author.

## Supplementary Materials

The following supporting information can be downloaded at: https://www.mdpi.com/article/10.3390/ma18245531/s1, Table S1: Structured summary of all engineered features used for model training.

## Nomenclature

ACC	Accuracy (14)—a classification performance metric representing the proportion of correctly predicted samples
CV	Cross-Validation—a statistical method for estimating the generalization performance of a machine–learning model
*k*–NN	*k-Nearest Neighbors*—a classification and regression algorithm based on the similarity between samples in the feature space
Ensemble (Bagging)	A ML approach that combines multiple decision trees trained on random subsets of data to reduce variance and improve prediction accuracy
R/T/P	Three microstructure generation protocols for non-overlapping circular inclusions described in detail in Section 3.2 and Part I
PCA	Principal Component Analysis—a linear dimensionality–reduction technique used to identify directions of maximal variance in data
*t*–SNE	*t*-distributed Stochastic Neighbor Embedding—a nonlinear method for projecting high-dimensional data into a low-dimensional space while preserving local neighborhood relationships
Permutation Importance	A model–agnostic metric that quantifies the relevance of a feature as the drop in model accuracy after random permutation of that feature
Feature Vector	A numerical representation of a sample containing selected descriptors (features) used for classification or regression
Structural sum	Complex–valued geometrical descriptors derived from the spatial arrangement of inclusions in composite materials
*f*	Concentration of inclusions, the area (volume) fraction of inclusions in RVE
RVE	Representative Volume Element—a representative unit cell of a composite structure used in homogenization and simulation analyses
η	Irregularity index (18) for a set of composites
χ	structural Irregularity measure (19) for a single microstructure
$z_{E}$	High–order energy—mean magnitude of structural-sum components over higher–index families
$z_{\phi }$	Phase chaos, the standard deviation of the arguments argz over selected structural–sum families
$z_{A}$	Im/Re asymmetry—ratio between the Euclidean norms of the imaginary and real components across selected structural-sum families
RSA	Random sequential adsorption protocol for simulation of non-overlapping disks
