# Supplementary material for: Computational Strategy for Analyzing Effective Properties of Random Composites–Part III: Machine Learning
Source: Materials (Basel). 2025 Dec 9;18(24):5531. doi: 10.3390/ma18245531 (PMC12735259; doi:10.3390/ma18245531)
Supplement: Supplementary file 1 [file materials-18-05531-s001.zip › materials-4004987-supplementary.pdf]

Supplementary Information for the article "Computational Strategy for Analyzing Effective Properties of Random Composites. Part III. Machine Learning

Table S1. Structured summary of all engineered features used for model training. The final feature matrix consists of approximately 130 standardized numerical features derived from the 14 complex structural sums listed in Eq. (12). Each feature group is shown together with its definition and the number of resulting variables.

Feature group	Definition / description	Count
Raw structural sums	Four basic descriptors extracted from each complex structural sum z_k: real part Re z_k, imaginary part Im z_k, magnitude $\|z_k\|$, and phase $\arg z_k$. These correspond directly to the 14 structural sums $E_1, \ldots, E_{14}$ used in Eq. (12).	$14 \times 4 = 56$
Family-level means	Family grouping based on structural-sum index patterns (e.g., 22, 33, 44, 223, 233, 333). For each family, the mean of Re z, $\|z\|$ and $\arg z$ is computed, summarizing intra-family geometric similarity and typical magnitude/phase behaviour.	≈ 18
Family-level standard deviations	Standard deviation of magnitudes $\|z\|$ and phases $\arg z$ within each structural-sum family. These descriptors reflect the degree of heterogeneity and local geometric disorder inside families of structurally related sums.	≈ 18
High-order energy metrics	Aggregated magnitude-based energy descriptors: total energy of all $\|z_k\|$, energy of higher-order sums (orders ≥ 3), and the normalized high-order energy component z_E used in the definition of the irregularity index η^*.	5
Phase-variability metrics	Angular-dispersion descriptors: standard deviation of $\arg z$ over selected families, normalized phase chaos z_ϕ, and inter-family angular spread. These metrics quantify phase randomness and angular irregularity.	5
Im/Re asymmetry metrics	Global and family-wise asymmetry descriptors: the ratio $\|\text{Im } z\|_2 / \|\text{Re } z\|_2$, the normalized asymmetry z_A, and contributions from different structural-sum families. They measure the imbalance between imaginary and real components in the complex plane.	6
Composite ratios and invariants	Feature combinations designed to be scale-robust or invariant: ratios of magnitudes between families, ratios Re $z_k / \|z_k\|$ and Im $z_k / \|z_k\|$, and normalized angular differences between families. These descriptors capture relative contrasts between structural levels.	≈ 12
Global summary statistics	Global summaries over the entire feature set: grand mean and variance of Re z, Im z, $\|z\|$ and $\arg z$, maximum magnitude and maximum phase deviation. They provide coarse characteristics of the overall complexity of each configuration.	10
**Total number of engineered features**	Sum over all groups above (depending on family sizes of structural sums).	≈ 130
